# A Hierarchical Multitask Learning Approach for the Recognition of Activities of Daily Living Using Data from Wearable Sensors

**DOI:** 10.3390/s23198234

**Published:** 2023-10-03

**Authors:** Muhammad Adeel Nisar, Kimiaki Shirahama, Muhammad Tausif Irshad, Xinyu Huang, Marcin Grzegorzek

**Affiliations:** 1 Department of Information Technology, University of the Punjab, Lahore 54000, Pakistan; 2 Department of Information Systems Design, Doshisha University, 1-3 Tatara Miyakodani, Kyotanabe 610-0394, Kyoto, Japan; kshiraha@mail.doshisha.ac.jp; 3 Institute of Medical Informatics, University of Lübeck, Ratzeburger Allee 160, 23562 Lübeck, Germany; m.irshad@uni-luebeck.de (M.T.I.); x.huang@uni-luebeck.de (X.H.); marcin.grzegorzek@uni-luebeck.de (M.G.); 4 Fraunhofer Research Institution for Individualized and Cell-Based Medical Engineering (IMTE), 23562 Lübeck, Germany

**Keywords:** activities of daily living, composite activity, atomic activity, machine learning, wearable sensors, hierarchical multitask learning

## Abstract

Machine learning with deep neural networks (DNNs) is widely used for human activity recognition (HAR) to automatically learn features, identify and analyze activities, and to produce a consequential outcome in numerous applications. However, learning robust features requires an enormous number of labeled data. Therefore, implementing a DNN either requires creating a large dataset or needs to use the pre-trained models on different datasets. Multitask learning (MTL) is a machine learning paradigm where a model is trained to perform multiple tasks simultaneously, with the idea that sharing information between tasks can lead to improved performance on each individual task. This paper presents a novel MTL approach that employs combined training for human activities with different temporal scales of atomic and composite activities. Atomic activities are basic, indivisible actions that are readily identifiable and classifiable. Composite activities are complex actions that comprise a sequence or combination of atomic activities. The proposed MTL approach can help in addressing challenges related to recognizing and predicting both atomic and composite activities. It can also help in providing a solution to the data scarcity problem by simultaneously learning multiple related tasks so that knowledge from each task can be reused by the others. The proposed approach offers advantages like improved data efficiency, reduced overfitting due to shared representations, and fast learning through the use of auxiliary information. The proposed approach exploits the similarities and differences between multiple tasks so that these tasks can share the parameter structure, which improves model performance. The paper also figures out which tasks should be learned together and which tasks should be learned separately. If the tasks are properly selected, the shared structure of each task can help it learn more from other tasks.

## 1. Introduction

Human activity recognition (HAR) plays a significant role in various fields like surveillance, medical sciences, and sports. The popularity and availability of wearable devices have turned wearable-based HAR into a promising research topic in machine learning and data science in recent years. Many machine learning approaches have been applied to build robust and generalized models for activity recognition [1,2,3]. Such models are called classification models, which are constructed using a training dataset made up of sensor data that have been labeled with the appropriate classes, such as activities. After that, the model is utilized to estimate the class of test data that are unknown.

To create an accurate model, researchers must first identify an appropriate abstracted representation of the data, known as “features”, that includes discriminant properties of the data relevant to the objective classification problem. This process is known as feature extraction. Previously, heuristic methods were used to build features based on prior knowledge of the sensor data for the target problem [4,5]. However, feature-learning algorithms have rapidly surpassed these methods in terms of automatically detecting relevant features in data. These algorithms, which employ neural networks or other machine learning models, determine which features are significant and relevant without the need for human intervention. They accomplish this objective by learning features from labeled examples in datasets without prior knowledge of the problem [6].

Deep learning, also known as machine learning with deep neural networks (DNNs), is one of the most popular feature-learning methods [7]. A DNN is composed of layers of artificial neurons that form an ensemble. Each neuron acts as a central computational unit characterized by its different internal parameters, such as weights and biases. When training a DNN, these parameters are fine-tuned to allow precise classification of the training data into their respective categories. Previous research has shown that neurons in well-trained DNNs encode unique features that outperform the capabilities of conventional human-evolved features. The effectiveness of DNNs in a variety of wearable computing applications has been demonstrated repeatedly over the years [3,8].

Deep learning techniques such as Convolutional Neural Networks (CNNs), Recurrent Neural Networks (RNNs), and Long Short-Term Memory Networks (LSTMs) are characterized by the automatic discovery of deep features that enable efficient categorization of unprocessed sensor data [9,10]. CNNs essentially function like feedforward neural networks. In contrast, RNNs differ from CNNs by introducing a directed cycle to represent dynamic temporal behavior, which gives them the ability to capture temporal relationships within time-series data. LSTM networks, on the other hand, improve RNNs by using more complex memory cells, effectively addressing the problem of the long-term dependence associated with standard RNNs.

Thus, DNNs not only learn the characteristic features from the raw sensor data but also perform classification to produce accurate results. However, learning robust features requires an enormous number of labeled data. Collecting a large dataset using wearable devices for problem-specific tasks is a difficult and challenging exercise. Most publicly available datasets focus on specific types of tasks related to their own problem domains. A dataset with diverse tasks that can be used as a general activity dataset is rare. Therefore, to implement a deep neural network, either we need to create our own large dataset or we can use the models trained on different datasets. Recently, transfer learning approaches have been used where models are pre-trained on existing datasets and then further trained on a smaller dataset for a particular problem to achieve good results on the latter [11]. However, it brings some challenges; e.g., the models need to be trained sequentially on different datasets, which increases the overall training time. Another major limitation in transfer learning is the problem of negative transfer [12]. Transfer learning is effective when the initial and target tasks have sufficient similarity during the initial training phase. Another challenge is to determine the appropriate number of training data for transfer learning to ensure that the model is not overfitted. Multitask learning (MTL) [13] is another option where multiple related tasks are learned simultaneously so that knowledge from each task can be reused by the others. MTL can be used to train smaller datasets to achieve robust performance. It takes advantage of the similarities and differences among multiple tasks so that they can share the same parameter structure, which improves the performance of the model. If the tasks are chosen appropriately, the shared structure of each task can facilitate more learning from other tasks.

This paper presents an MTL technique that employs the combined structure of CNN and LSTM to recognize activities of daily living (ADLs) like brushing teeth, cleaning a room, or preparing a meal. As we know, atomic activities function as components of ADLs or composite activities; e.g., when cleaning a room, a person mainly walks, bends, and squats to clean the floor with a mop and sometimes stands to clean surfaces such as windows and tables. On the other hand, the characteristics of composite activities may reflect the relationships between atomic activities and help further in the recognition of atomic activities. Therefore, the proposed MTL technique aims to recognize atomic and composite activities jointly. In recognizing atomic and composite activities, MTL can discover the similarities between these two types of activities that are difficult to learn with single-task learning (STL). At the same time, the differences between atomic and composite activities are a kind of inductive bias that can help improve the generalization capabilities of atomic and composite activity recognition models.

We provide the following scientific contributions:

- The main contribution is presenting a novel architecture for multitask learning for tasks with different temporal scales. They are incorporated into one multitask learning framework.
- Multitask learning does not always increase performance. Therefore, it is important to know which tasks should be learned together to yield better results than single-task learning. We performed extensive experiments and identified such *cooperating* tasks that should be learned together, and *competing* tasks that should be learned separately.
- The third contribution is to present the improved results of atomic and composite activities of CogAge datasets as compared to the previously produced results on these datasets.

This paper is organized as follows: Section 2 presents the highly relevant existing research regarding HAR and MTL. Section 3 provides a brief overview of the proposed MTL approach. Section 4 provides a detailed description of all STL and MTL methods proposed in this paper. Section 5 describes the complete evaluation procedure of the proposed approach, including the description of the datasets, the presentation of the experiments performed on the datasets, and the results to evaluate the proposed approach against other state-of-the-art techniques. It also provides a discussion on the outcome of the experiments. Finally, Section 6 provides a conclusion.

## 2. Related Work

In this section, we provide an overview of existing research that is highly relevant to the proposed approach. We begin by mentioning human activity-recognition models and then discuss approaches that use multitask learning.

### 2.1. Human Activity Recognition

HAR has made significant research advances in recent years. Many HAR studies, as summarized in Table 1, are concerned with the recognition of atomic activities. For example, Gupta et al. [14] and He et al. [15] used a triaxial accelerometer to recognize atomic activities such as “walking” “sitting”, and “jumping”, whereas Lara et al. in [16] used a smartphone and a chest strap to recognize similar atomic activities.

In addition to atomic activity recognition, many HAR studies also address the recognition of composite activities. Composite activity recognition has been attempted in two ways. The first approach did not differentiate between composite activities and atomic activities, employing identical techniques originally intended for the recognition of atomic activities. As Dernbach et al. [17] used motion sensors such as accelerometers and gyroscopes to detect atomic activities such as “standing” and “sitting”, they also detected composite activities like “cleaning kitchen” and “cooking”. Bao et al. [18] employed a set of five biaxial accelerometers to identify a spectrum of twenty human activities. Some of them are atomic activities like “walking” and “running”, while others are composite activities such as “working” and “eating”. Their results showed that the recognition accuracy varied significantly between atomic activities and composite activities.

Given the complex structure of composite activities, existing methods for identifying atomic activities struggle to categorize them effectively. Therefore, many researchers used the second approach to recognize composite activities where HAR has been presented in the form of multilevel frameworks. Nisar et al. [19] proposed a multilevel framework to recognize activities of daily living. At the first level of their framework, atomic activities are recognized using the codebook [20] approach, and then the recognition scores of atomic activities are used to detect composite activities employing the rank pooling approach at the second level of their framework. In addition, these approaches [21,22,23,24,25,26,27] also present hierarchical models where atomic activities are recognized at the first level and then the composite activities that can be seen as the combinations of atomic activities are detected at the higher level. In our proposed approach, we also present a multi-level architecture. However, some of the above methods treated HAR as a single-label classification task. They did not consider the correlation between atomic and composite activities, which could improve the performance of their HAR models. In this paper, state-atomic, behavioral-atomic, and composite activity recognition are considered as three related tasks. They are learned together, and what is learned for one task can help the others be learned better.

Researchers have also implemented deep learning methods for HAR. For example, Zeng et al. [28] used a CNN to extract characteristic features for HAR that allow it to capture local patterns and maintain consistency of activity data across different scales. Another approach presented by Yang [29] employed a deep CNN for automated feature extraction from time-series data, enabling recognition of human activity and hand gestures. Guan et al. [30] presents LSTM networks for HAR using time-series data. Their model used various LSTM networks fused together to improve HAR performance. In the study presented by Morales et al. [31] related to multimodal human activity detection based on wearable sensors, they used deep convolutional and LSTM networks. Their research demonstrated the superior detection accuracy achieved by combining CNN and LSTM networks compared to using CNN or LSTM alone and served as inspiration for our approach. Hammerla et al. [32] examined how various deep learning techniques, including deep feed-forward networks, CNNs, and bi-directional LSTM networks, affected human activity recognition (HAR). Dua et al. [33] presented a multi-input CNN and Gated Recurrent Unit (GRU)-based activity-recognition approach and produced adequate results. Challa et al. [34] and Sakorn et al. [35] also implemented approaches based on multi-branch CNN and LSTM to recognize physical activities.

All of these studies demonstrated the effectiveness of deep learning in HAR. Nevertheless, a common challenge encountered in these studies was that deeper networks tended to provide superior performance but were more difficult to converge. In order to address this issue, we have employed a multi-branch architecture that is less complex and easier to train than the aforementioned models. Notably, this method can achieve equivalent or even better performance than deeper networks.

**Table 1:** Comparison of different approaches to recognize human activities, particularly activities of daily living (ADLs).

Comparison of Human Activity Recognition Approaches						
S. No.	Activity Type	Num. of Activities	Multilevel	Data Acq. Sensor(s)	Features	Ref.
1.	atomic	6	no	1 acc.	handcrafted	Gupta [14]
2.	atomic	4	no	1 acc.	handcrafted	He [15]
3.	atomic	5	no	1 acc, phys.	handcrafted	Lara [16]
4.	atomic, comp.	9, 7	no	smartphone	handcrafted	Dernbach [17]
5.	atomic	20	no	4 acc	handcrafted	Bao [18]
6.	atomic, comp.	43	no	RFID, motion sensors, camera, mic	handcrafted	Logan [21]
7.	atomic	11	no	3 acc., 3 gyro.	handcrafted	Bulling [23]
8.	atomic	34	no	2 acc.	handcrafted	Tam [24]
9.	atomic, comp.	4, 5	yes	smartphone	handcrafted	Liu1 [25]
10.	atomic, comp.	18, 4	yes	IMUs	handcrafted	Liu2 [26]
11	atomic, comp.	8, 9	yes	smartphone, smartwatch, chest-starp phys.	handcrafted	Peng [27]
12.	atomic	18	no	IMUs	CNN	Zeng [28]
13.	atomic	18	no	IMUs	CNN	Yang [29]
14.	atomic	18	no	IMUs	LSTM	Guan [30]
15.	atomic	18	no	IMUs	CNN + LSTM	Morales [31]
16.	atomic	18	no	IMUs	CNN + LSTM	Hammerla [32]
17.	atomic	12	no	IMUs	CNN + GRU	Dua [33]
18.	atomic	12	no	IMUs	CNN + BiLSTM	Challa [34]
19.	atomic	6	no	IMUs	CNN + LSTM	Sakorn [35]
20.	atomic, comp.	61, 7	yes	smartphone, smartwatch, smartglasses	CB + RP	Nisar [19]
21.	atomic, comp.	6, 55, 7	yes	smartphone, smartwatch, smartglasses	CNN + LSTM	Proposed Approach

Acq: Acquisition; comp: composite; acc: accelerometer; gyro: gyroscope; phys: physiological sensors; CB: codebook approach; RP: rank pooling approach.

### 2.2. Multitask Learning

Multitasking learning refers to a method of using the common structure of a model to accomplish multiple tasks simultaneously [13]. When tasks are chosen wisely, this shared structure improves the model’s ability to generalize. Multitask learning has produced impressive results in areas such as speech recognition, text analysis, and computer vision.

In computer vision, Zhang et al. [36] have fine-tuned the optimization of facial feature recognition along with different but subtly related tasks such as head pose estimation and facial feature inference. Another approach [37] used a multitasking CNN architecture to jointly perform face detection and alignment. Multitask learning has been implemented to enhance the performance of long-term activity-recognition models by leveraging the need for an enormous number of labeled data [38]. The learned model simultaneously predicts activity and recognizes the start and end times of each action instance from unedited video.

Li et al. [39] proposed a framework for multitask human activity recognition that takes into account not only activity but also the wearer’s identity and gender and the sensor’s position on the body. They used handcrafted features, encompassing characteristics in both the temporal and frequency domains of the original data. Classification was conducted through a multitask learning framework comprising a fully connected network and a CNN. In contrast to their approach, we do not use handcrafted features and instead automatically learn features for both atomic and composite activities.

Chen et al. [40] presented a deep multitask learning framework called METIER, which recognizes a human activity along with the user who performed that activity. Their model jointly trains activity- and user-recognition models by employing CNN and bi-LSTM networks on fixed-length segments of sensor data. In contrast to their approach, our multitask learning model is learned for activities with different temporal lengths.

AROMA [41] is a framework for HAR that is closely related to our approach. The authors presented models that used a CNN-LSTM-based architecture to recognize simple and complex activities. They have jointly trained both types of activities using multitask learning. However, our approach is different from theirs in many ways; for example, ours learns three different tasks. Also, we do not fix the lengths of complex activities. We present a single architecture for the activities of different temporal scales.

MTL is found to enhance the performance of shared networks compared to single-task networks by solving multiple tasks together. Standley et al. [42] noted that multitasking can theoretically offer other benefits as well, such as improving task-prediction accuracy and reducing training and inference time by requiring only a single network to be trained and evaluated rather than using separate networks for independent tasks, as well as increased data efficiency. However, this is not always the case, as multitask performance can suffer to the point that single-task networks are superior for individual tasks. This phenomenon could be due to different learning rates on different tasks or due to one task exerting a dominant influence on the learning process, which then negatively affects performance on other tasks. Such tasks are considered to be competing tasks. On the other hand, if the objectives of the tasks do not conflict, joint training can result in maintaining or even improving performance on both tasks. Such tasks are referred to as cooperating tasks. Intuitively, the advantages or disadvantages of using multitask learning seem to depend on the relationship between the jointly trained tasks. Therefore, the authors in [42] addressed the question of which tasks should be learned using the multitask approach and which tasks would be better learned independently by proposing a framework that can empirically examine the relationships among the tasks.

In the proposed multitask approach, we also empirically study these relationships among different types of activities and propose the method that learns cooperating tasks together and competing tasks independently. Table 2 presents a summary of the aforementioned MTL approaches.

**Table 2:** Comparison of different multitask learning approaches.

Comparison of Multitask Learning (MTL) Approaches								
S. No.	Data	No. of Tasks	Tasks Type	Classes per Task	Len.	Datasets in MTL*	MTL Arch.	Ref.
1.	images	4	h.pose, gen, sm, gl	3, 2, 2, 2	fixed	1	shared	Zhang et al. [36]
2.	video	2	act, LCE	17	variable	1	shared	Li [38]
3.	sensors	4	act, user, gen, pos	8, 15, 2, 7	fixed	1	shared	Li [39]
4.	sensors	2	act, user	17, 33	fixed	1	shared	Chen et al. [40]
5.	sensors	2	s.act, c.act	34, 4	fixed	1	shared	AROMA [41]
6.	sensors	3	st, bhv, comp	6, 55, 7	variable	3	shared for comp*	Proposed Approach

S. No.: serial number; Len: length of an example; Arch: architecture; h.pose: head pose; gen: gender; sm: smiling; gls: wearing glasses; act: activity; pos: position of sensor; s.act: simple activity; c.act: complex activity; st: state; bhv: behavioral; comp: composite; Datasets in MTL*: number of datasets trained together; MTL Arch.: Multitask learning architecture; shared for comp*: shared architecture for composite activity examples but separate channels for state and behavior activities; variable: composite activity length is not fixed.

## 3. Overview of the Proposed Approach

**Figure 1:**
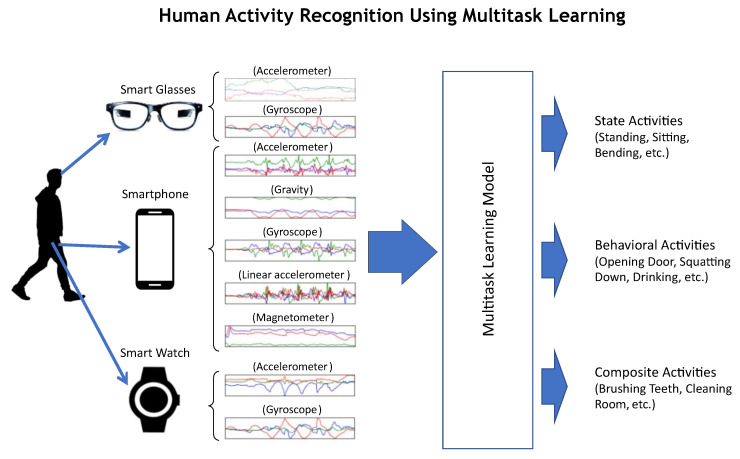
A multitask learning approach for human activity recognition using multimodal sensor data acquired from wearable devices.

The proposed multitask architecture aims to learn three recognition tasks, i.e., state, behavioral, and composite activities, as shown in Figure 1. Multiple approaches have been tested to achieve the objective of learning the aforementioned tasks with good accuracy.

**Figure 2:**
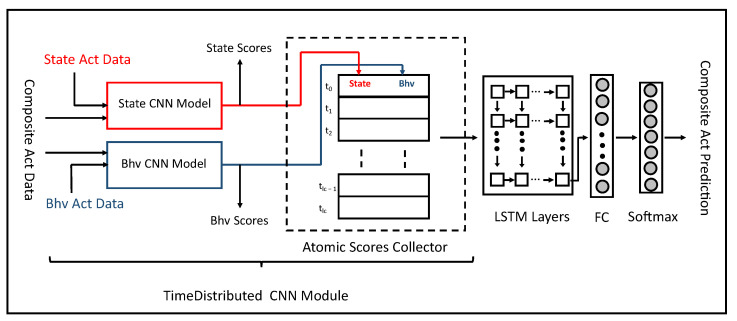
Overview of the multitask learning architecture to learn state, behavioral, and composite activities together.

An overview of the proposed architecture that produces the best results for all types of activities is shown in Figure 2. It contains two major parts: time-distributed CNN modules and LSTM layers. The time-distributed CNN contains two sub-networks of multi-branch CNN architecture for state and behavioral activities. These two sub-networks are referred to as “State CNN Model” (SCM) and “Bhv CNN Model” (BCM). Each model consists of a multibranch CNN module that contains two pairs of convolutional and pooling layers for the input data of a sensor. The output of the last pooling layer of each sensor module is concatenated and first sent to the fully connected layers and then to a softmax layer that produces the output for each of the atomic activities.

The second part of the multitask architecture consists of LSTM and fully connected layers. This part is used to recognize composite activities. It is important to note that the atomic activity instances consist of shorter and fixed-length movements, whereas composite activity instances have longer and variable lengths. The proposed approach uses a hierarchical model to recognize composite activities. The atomic activities are trained using their respective multibranch CNN architectures (SCM and BCM), which are also responsible for generating the atomic scores. Composite activities are trained using a combined architecture of the time-distributed multibranch CNN and LSTM and fully connected layers.

The LSTM layers of the model receive input from the combined CNN part and encode the temporal relationship within an example of a composite activity. The fully connected and softmax layers are finally used for the prediction of a composite activity.

The model is trained using the following three main steps:

- In the first step, sensor data of the state activities are sent to SCM (outlined with red color in Figure 2), which generates state atomic scores. The loss is calculated, and the gradients of the loss with respect to the model parameters are back-propagated to optimize the training model of the state activities. The weights of SCM are updated in this step.
- In the second step, the sensor data of behavioral activities are sent to BCM (outlined with blue color in Figure 2), which generates behavioral atomic scores. The loss is calculated using the behavioral activity labels and back-propagated to update the weights of BCM.
- In the third step, each composite activity example is divided into fixed-length segments. It is important to note that the number of segments is not fixed but depends on the original length of the composite activity example. These segments are sent to SCM and BCM to generate the state and behavioral atomic scores. The time distribution layer gathers the atomic scores of all segments (atomic scores collector) and then passes them to the LSTM layers. The LSTM layers receive the time-distributed atomic scores of all segments of a composite activity instance and finds the temporal relationship between them. The output sequence of the last layer is first sent to a fully connected and then a softmax layer that generates recognition scores for each composite activity. The loss function computes the gradients of the loss and performs backpropagation to update the weights of the entire model for optimization.

This procedure is repeated in the consequent epochs during the training phase. The proposed multitask architecture yields outstanding recognition scores as compared to the single-tasking models for each of the three activities.

## 4. Methodology

**Figure 3:**
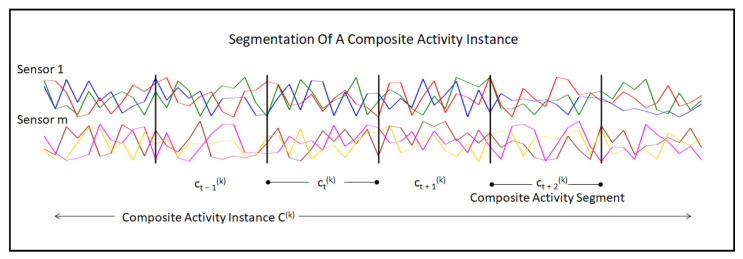
The figure shows the segmentation of a composite activity instance $C^{\left(k\right)}$. Two of the sensor modalities $s_{1}$ and $s_{m}$ are shown in the figure. The horizontal axis of the graph represents the time. The equal-size segments of the composite activity instance are represented by $c_{i}^{\left(k\right)}$.

This section gives a detailed methodological description of our proposed approaches that aim to recognize *M* atomic and *N* composite activities. We begin with the following definitions:

- **Definition 1** *Atomic activities* (A) are simple and fixed-length (la) human activities like “standing”, “walking”, and “opening door”. Atomic activities are divided into two categories, state activities (S) and behavioral activities (B), such that S⊂A and B⊂A. We have $M_{S}$ and $M_{B}$ labels for state and behavioral activity, respectively, such that $M=M_{S}+M_{B}$. Assume that we have $K_{A}$ instances of atomic activities (Equation (1)), where each instance consists of *q* sensor modalities. The *k*th atomic activity instance $A^{\left(k\right)}$ ($1\leq k\leq K_{A}$) is described in Equation (2). State and behavioral activities also exhibit the same structure as mentioned in Equation (3).
  (1) $$A=\{A^{\left(1\right)},A^{\left(2\right)},\cdots ,A^{\left(K_{A}\right)}\}$$
  (2) $$A^{\left(k\right)}=\left(s_{1}^{\left(k\right)},s_{2}^{\left(k\right)},\cdots ,s_{q}^{\left(k\right)}\right)$$
  (3) $$A^{\left(k\right)}=\left\{\begin{array}{c} S^{\left(k\right)}\\ B^{\left(k\right)} \end{array}\right.$$
- **Definition 2** *Sensor modality* ($s_{m}$) represents the raw sensor data stream of a sensor *m*, where 1≤m≤q and m∈{sp−acc,sp−gyr,sp−grav,sp−linAcc,sp−magn,sw−acc,sw−gyro,sg−acc,sg−gyr}, which yields that q=9. Each sensor modality has 3 sensor channels, i.e., x, y, and z axes. The data of each sensor modality have their own length $l_{m}$ depending upon the sampling rate of each sensor *m*. The length $l_{m}$ of each sensor modality $s_{m}$ is fixed across all instances of atomic activities. Equation (4) describes the sensors’ modalities at different time points, whereas Equation (5) shows the values of three sensor channels at a time point *t*,
  (4) $$s_{m}=\left(s_{m,1},s_{m,2},\cdots ,s_{m,t},\cdots ,s_{m,l_{m}}\right)$$
  (5) $$s_{m,t}={\left(s_{m,t,x},s_{m,t,y},s_{m,t,z}\right)}^{T}$$
- **Definition 3** *Composite activities* (Cs) are long-term and complex human activities such as “brushing teeth”, “cleaning room”, and “styling hair”. Assume that we have $K_{C}$ instances (as described in Equation (6)) of *N* composite activity classes where each instance has its own length. Mathematically, the *k*th composite activity instance $C^{\left(k\right)}$ ($1\leq k\leq K_{C}$) has a length of $T_{k}$ time points. The data of composite activities also consist of the sensor modalities described in Definition 2.
  (6) $$C=\{C^{\left(1\right)},C^{\left(2\right)},\cdots ,C^{\left(K_{C}\right)}\}$$
- **Definition 4** *Composite activity segments* ($c^{\left(k\right)}$) are fixed-length segments of an instance of the composite activity. The number of segments $lc^{k}$ of any instance *k* of a composite activity can differ depending on the length $T_{k}$ of $C^{\left(k\right)}$. An instance of a composite activity in terms of fixed-length segments can be described in Equation (7). Figure 3 shows the graphical representation of these segments.
  (7) $$C^{\left(k\right)}=\left(c_{1}^{\left(k\right)},c_{2}^{\left(k\right)},c_{3}^{\left(k\right)},\cdots ,c_{lc^{k}}^{\left(k\right)}\right)$$
- **Definition 5** *Tasks for atomic activity recognition*: Given an atomic activity instance $A^{\left(k\right)}$ and its atomic activity label $y_{A}^{\left(k\right)}$, the goal is to find a mapping function $f_{A}:A^{\left(k\right)}\to y_{A}^{\left(k\right)}$, and the predicted label $f_{A}\left(A^{\left(k\right)}\right):y_{A}^{{\left(k\right)}_{p}}$ should be the actual label.
- **Definition 6** *Task for state atomic activity recognition*: Given a state atomic activity instance $S^{\left(k\right)}$ and its state activity label $y_{S}^{\left(k\right)}$, the goal is to find a mapping function $f_{S}:S^{\left(k\right)}\to y_{S}^{\left(k\right)}$, and the predicted label $f_{S}\left(S^{\left(k\right)}\right):y_{S}^{{\left(k\right)}_{p}}$ should be the actual label.
- **Definition 7** *Task for behavioral atomic activity recognition*: Given a behavioral atomic activity instance $B^{\left(k\right)}$ and its behavioral activity label $y_{B}^{\left(k\right)}$, the goal is to find a mapping function $f_{B}:B^{\left(k\right)}\to y_{B}^{\left(k\right)}$, and the predicted label $f_{B}\left(B^{\left(k\right)}\right):y_{B}^{{\left(k\right)}_{p}}$ should be the actual label.
- **Definition 8** *Task for composite activity recognition*: Given a composite atomic activity instance $C^{\left(k\right)}$ and its composite activity label $y_{C}^{\left(k\right)}$, the goal is to find a mapping function $f_{C}:C^{\left(k\right)}\to y_{C}^{\left(k\right)}$, and the predicted label $f_{C}\left(C^{\left(k\right)}\right):y_{C}^{{\left(k\right)}_{p}}$ should be the actual label.
- **Definition 9** *Task for the joint recognition of state and behavioral atomic activities*: Given a set of activity instances and its corresponding state and behavioral atomic activity labels, a state activity classifier $f_{S}$ and a behavioral activity classifier $f_{B}$ are jointly trained by minimizing total loss $L\left(f_{S}\right)+L\left(f_{B}\right)$, where $L(f_{S})$ and $L(f_{B})$ denote the loss functions of $f_{S}$ and $f_{B}$, respectively.
- **Definition 10** *Task for the joint recognition of state, behavioral and composite activities*: Given a set of activity instances and its corresponding state, the behavioral and composite activity labels are for a multi-branch joint classifier in which $f_{S}$ and $f_{C}$ are trained together in the first branch by minimizing total loss $L\left(f_{S}\right)+L\left(f_{C}\right)$, and the second branch trains $f_{B}$ and $f_{C}$ by minimizing total loss $L\left(f_{B}\right)+L\left(f_{C}\right)$, where $L(f_{C})$ denotes the loss function of $f_{C}$.

### 4.1. Single-Task Learning for Atomic Activities

Our approach uses CNN to extract features from the instances of atomic activities. We first briefly introduce CNN and then explain how our approach employs it.

A CNN consists of input, output, and multiple hidden layers. Each layer contains multiple units that are used for processing the data. The data are processed layer by layer, where the output of the previous layer is used as input to the next layer.

The input layer receives activity data collected using wearable devices involving several sensor modalities $s_{m}$. Data are then forwarded to the hidden layers for further processing. Hidden layers are either convolutional, pooling, or fully connected (dense) layers.

The convolutional layers extract features to provide the abstract representation of the input data. It convolves the data received from the previous layer by employing several convolutional kernels to generate feature maps. Equation (8) represents the sensor modalities provided to the first convolutional layer. For the sake of simplicity, we take three time points, for example, $s_{m,t-1},s_{m,t},$ and $s_{m,t+1}$, to form a segment $f_{t}^{\left(1\right)}$ as input to the first convolutional layer. Equation (9) explains the convolution operation to produce a feature map at time point *t* for a layer *l*. Equation (10) describes the feature maps convolved over all time points using one of the kernels for a layer *l*.

(8) $$\begin{array}{c} s_{m}=\left(s_{m,1},s_{m,2},\cdots ,\underset{s_{m,t}=f_{t}^{\left(1\right)}}{\underset{︸}{s_{m,t-1},s_{m,t},s_{m,t+1}}}s_{m,t-1},\overset{s_{m,t+1}=f_{t+1}^{\left(1\right)}}{\overset{︷}{s_{m,t},s_{m,t+1},s_{m,t+2}}}s_{m,t},s_{m,t+1},s_{m,t+2},\cdots ,s_{m,l_{m}}\right) \end{array}$$

(9) $$f_{j,t}^{\left(l+1\right)}=\sigma \left(K_{j}^{\left(l\right)}f_{t}^{\left(l\right)}+b_{j}^{\left(l\right)}\right)$$

(10) $$f_{j}^{\left(l+1\right)}=\left(f_{j,1}^{\left(l+1\right)},f_{j,2}^{\left(l+1\right)},\cdots ,f_{j,t}^{\left(l+1\right)},f_{j,t+1}^{\left(l+1\right)},\cdots ,f_{j,l_{m}-1}^{\left(l+1\right)}\right)$$

In these equations, $f_{j,t}^{\left(l+1\right)}$ represents the value of the jth feature map at time point *t* in layer l+1, $f_{j}^{\left(l+1\right)}$ represents jth feature map of all time points in layer l+1, $K_{j}^{\left(l\right)}$ represents the convolutional filter convolved over the input $f_{t}^{\left(l\right)}$ to generate the jth feature map in layer (l+1), $b_{j}^{\left(l\right)}$ is a bias and σ() represents the activation function to introduce non-linearity in the output. For a layer *l*, we use $F^{\left(l\right)}$ convolutional filters such that $1\leq j\leq F^{\left(l\right)}$. Therefore, each convolutional layer outputs $F^{\left(l\right)}$ feature maps.

The pooling layer performs non-linear down-sampling by implementing a pooling function, e.g., maximum and average. It decreases the spatial size of the representations and also reduces the number of parameters in order to make the outputted features more robust to variations in the temporal positions of the input data. Equations (11) and (12) explain the operation of maximum and average pooling, respectively.

(11) $$f_{j,t}^{\left(l+1\right)}=\max_{i=1}^{p}\left(f_{j,t\times p+i}^{\left(l\right)}\right)$$

(12) $$f_{j,t}^{\left(l+1\right)}=\frac{1}{p}\sum_{i=1}^{p}\left(f_{j,t\times p+i}^{\left(l\right)}\right)$$

In these equations, $f_{j}^{\left(l\right)}$ represents the value of the *j*th unit in layer *l*, and *p* denotes the size of the pooling region.

**Figure 4:**
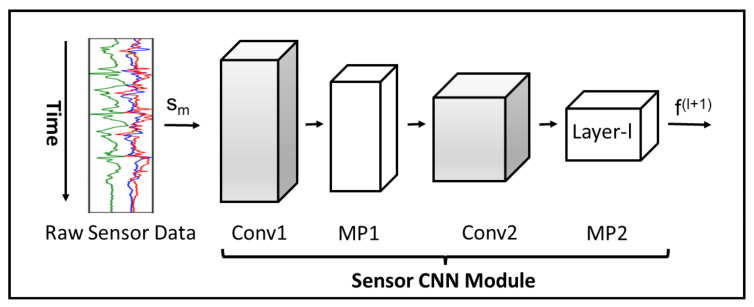
The sensor module represents a CNN branch of our proposed model. The branch receives raw sensor data $s_{m}$ of a sensor *m* for convolutional and max pooling layers. Each layer *l* receives a feature map $f^{\left(l\right)}$ as input and produces an output feature map $f^{\left(l+1\right)}$ for the next layer.

**Figure 5:**
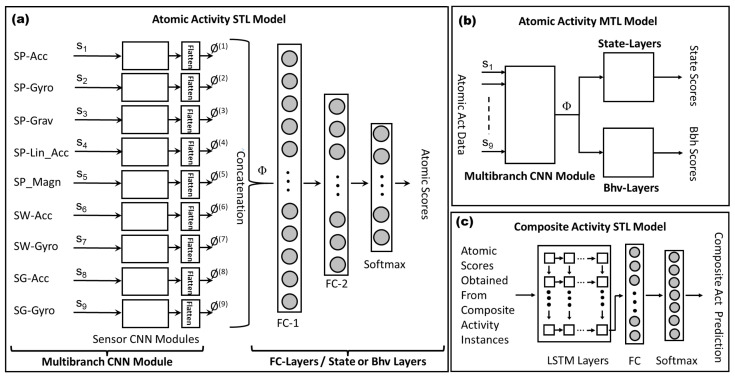
The figure shows the architectures of three models. The first figure (a) shows the multibranch architecture for the single-task learning (STL) model of atomic activities. State and behavioral activities use this model to generate atomic scores. The second figure (b) is the architecture of the multitask learning (MTL) model for atomic activities. They share the multibranch CNN module; however, they exclusively use their own state and behavioral (bhv) modules. The third figure (c) represents the architecture of the single-task learning model for composite activities. The instances of composite activities are first sent to either STL or MTL atomic models to generate atomic scores. Then, these scores are provided to composite activity STL model for the prediction of the labels.

Figure 4 and Figure 5a show the CNN architecture for single sensor and multibranch modules, respectively. We implement two convolutional layers to generate feature maps from sensor data. After each convolutional layer, we employ a maximum pooling layer to reduce the network size and control overfitting. The convolution and maximum pooling operations can be performed either on each sensor modality independently or across all sensor modalities. In the proposed approach, the convolution and maximum pooling operations are performed along the time axis, which keeps each sensor channel independent in CNN and prevents the data-compatibility issues caused by the fusion of sensor data with different sampling rates. To introduce nonlinearity, we use the rectified linear unit (ReLU) as an activation function in each convolutional layer. Equation (13) explains the ReLU operation on a feature map $f_{j}$.

(13) $$\sigma \left(f_{j,t}\right)=\max\left(f_{j,t},0\right)$$

The output of the last pair of convolutional and pooling layers at each branch of CNN is flattened into a vector sequence ($\phi^{\left(m\right)}$), where the time axis remains and the other axes are flattened.

(14) $$\overset{\mathrm{Output}\;\mathrm{of}\;\mathrm{last}\;\mathrm{pooling}\;\mathrm{layer}}{\to }\left[\begin{array}{c} f_{1,1},f_{1,2},\cdots ,f_{1,C}\\ f_{2,1},f_{2,2},\cdots ,f_{2,C}\\ \vdots \\ f_{R,1},f_{R,2},\cdots ,f_{R,C} \end{array}\right]\overset{\mathrm{Flattening}}{\to }\begin{array}{c} \phi^{\left(m\right)}= \end{array}\left[\begin{array}{c} f_{1,1}\\ f_{1,2}\\ \vdots \\ f_{1,C}\\ f_{2,1}\\ f_{2,2}\\ \vdots \\ f_{R,C} \end{array}\right]$$

The flattened vectors ($\phi^{\left(m\right)}$) are then concatenated to form a big joint vector Φ for all sensor modalities, as described in Equation (15):

(15) $$\begin{array}{c} \begin{array}{c} \Phi = \end{array}\left[\begin{array}{c} \phi^{\left(1\right)}\\ \phi^{\left(2\right)}\\ \vdots \\ \phi^{\left(q\right)} \end{array}\right] \end{array}$$

The vector Φ is forwarded to the fully connected layer to produce output vector z, as described in Equation (16).

(16) z=WΦ+b

where vector z contains unnormalized log probabilities and W is the weight matrix.

We use three fully connected layers in our model and the output of the last fully connected layer serves as the input to a softmax function. The softmax function converts a vector of numbers into a vector of probabilities, where the probabilities of each value are proportional to the relative scale of each value in the vector z as shown in Equation (17),

(17) $$q_{A}=\mathrm{softmax}\left(z\right)=\left(\frac{\exp(z_{1})}{\sum_{j=1}^{M}\exp\left(z_{j}\right)},\frac{\exp(z_{2})}{\sum_{j=1}^{M}\exp\left(z_{j}\right)},\cdots ,\frac{\exp(z_{M})}{\sum_{j=1}^{M}\exp\left(z_{j}\right)}\right)$$

where $q_{A}$ contains the atomic scores for an atomic activity instance and exp represents the exponent function. We have also used the softmax function as a classifier that predicts the atomic activity label, as shown in Equation (18):

(18) $$f_{A}\left(z\right)=P\left(y_{A}^{p}=y_{A}|z\right)=\frac{\exp(z_{y_{A}})}{\sum_{j=1}^{M}\exp\left(z_{j}\right)}$$

where z is the output of the last fully connected layer, which is sent to the softmax function, $y_{A}$ is the label of the atomic activity, $y_{A}^{p}$ is the label predicted by the classifier $f_{A}$, *M* is the number of atomic activity labels, and $z_{j}$ refers to the jth element of unnormalized log probability vector z. The predicted label is assigned to the one with the highest probability, i.e., $y_{A}\leftarrow \arg\max_{y_{A}=1}^{M}q_{A}$.

### 4.2. Multitask Learning for Atomic Activities

Multitask learning for atomic activities is achieved by constructing a joint model (as shown in Figure 5b) for state and behavioral activities where the multibranch CNN architecture is shared between them, and then the output vector Φ is sent to the state and behavioral modules. These modules consist of fully connected layers, as shown in Figure 5a.

Let $f_{S}\left(S^{\left(k\right)}\right)$ and $f_{B}\left(B^{\left(k\right)}\right)$ be two classifiers on state activity instance $S^{\left(k\right)}$ and behavioral activity instance $B^{\left(k\right)}$, respectively. Both of the classifiers use the softmax function described in Equation (17) for classification. The respective softmax functions return the probability vectors $q_{S}$ and $q_{B}$ for state and behavioral activities, respectively. The cross-entropy loss function is used by these classifiers. The loss function $L(f_{S})$ computes the loss for state activities, as shown in Equation (19),

(19) $$L\left(f_{S}\right)=-\frac{1}{U}\sum_{u=1}^{U}\left(\sum_{m=1}^{M_{S}}y_{m}^{\left(u\right)}\log\left(\frac{\exp(z_{m}^{\left(u\right)})}{\sum_{j=1}^{M_{s}}\exp\left(z_{j}^{\left(u\right)}\right)}\right)\right)$$

The loss function $L(f_{S})$ can also shown in a compact form in Equation (20). The other loss function $L(f_{B})$ is defined for behavioral activities in Equation (21)

(20) $$L\left(f_{S}\right)=-\frac{1}{U}\sum_{u=1}^{U}{y_{S}^{\left(u\right)}}^{T}\log\left(q_{S}^{\left(u\right)}\right)$$

(21) $$L\left(f_{B}\right)=-\frac{1}{U}\sum_{u=1}^{U}{y_{B}^{\left(u\right)}}^{T}\log\left(q_{B}^{\left(u\right)}\right)$$

The joint loss of the atomic activity classifier $L(f_{A})$ is computed as,

(22) $$L\left(f_{A}\right)=w_{S}\cdot L\left(f_{S}\right)+w_{B}\cdot L\left(f_{B}\right)$$

where $w_{S}$ and $w_{B}$ are the weights to balance $L(f_{S})$ and $L(f_{B})$. The goal of the proposed multitask approach is to train the model by minimizing the joint loss function as follows:

(23) $$f_{S},f_{B}\leftarrow \arg\min\;L\left(f_{A}\right)$$

The classification functions $f_{S}\left(S;\theta_{S}\right)$ and $f_{B}\left(B;\theta_{B}\right)$ have a set of parameters $\theta_{S}$ and $\theta_{B}$ that should be minimized according to the loss function $L(f_{A})$.

(24) $${\theta_{S}}^{*},{\theta_{B}}^{*}\leftarrow \underset{\theta_{S}\theta_{B}}{\text{arg}\;\text{min}}\;L\left(f_{A}\right)$$

After training, we obtain the optimized set of parameters ${\theta_{S}}^{*}$ and ${\theta_{B}}^{*}$ in the functions $f_{S}\left({S;\theta_{S}}^{*}\right)$ and $f_{B}\left({B;\theta_{B}}^{*}\right)$, respectively.

Training a multitask model involves the backpropagation process, in which the loss generated by the classification task of one type of atomic activity also affects the parameters of the shared layers of the model of both activities. However, the process continues unless we obtain the optimal set of parameters ${\theta_{S}}^{*}$ and ${\theta_{B}}^{*}$ with a minimum value of the joint loss.

### 4.3. Single-Task Learning for Composite Activities

We use an LSTM network for composite activity detection. LSTM networks are an evolution of RNNs, which belong to a category of artificial neural networks characterized by cyclic connections between neurons. This structural design makes the output of the neurons dependent on the state of the network in the previous time steps and allows them to store information from past data. This special property empowers RNNs to recognize patterns with extended dependencies and capture the temporal context of input data.

RNNs are susceptible to the challenge of *vanishing* or *exploding* gradients, which occur when the derivatives of the error function with respect to the network weights become extremely small or excessively large during the training process. In both scenarios, the ability of the backpropagation algorithm to update the weights is compromised. To solve this problem, an alternative to the standard neuron, known as the Long Short-Term Memory (LSTM) cell, was introduced [43]. The LSTM cell is specifically designed to retain information over time by storing it in internal memory and making updates, outputs, or deletions based on the input and the state of the previous time step.

This mechanism, depicted in Figure 5c, is achieved by first generating state and behavioral atomic scores for the segments of a composite activity instance. These segments are sent to either the STL or MTL models of atomic activities to produce atomic scores. The behavioral and state scores of a segment at time *t* are joined to form an input vector $v_{t}$ to LSTM layers. The following equations delineate a set of internal computational components called *gates*, each with its own weights, biases, and activation functions. These gates include the input gate, the output gate, and the forget gate, which serve different purposes. The input gate controls the input to the cell and preserves its memory $c_{t}$; the output gate regulates the output of the cell and prevents it from interfering with the computation of $c_{t+1}$, and the forget gate ensures that the cell’s internal memory is cleared at time *t*. All of these gates operate on the cell’s input vectors $v_{t}$ at time *t* and the cell’s output from the previous time step, $h_{t-1}$, according to the following equations:

(25) $$\begin{array}{c} y_{t}^{i}=\sigma \left(W_{i}v_{t}+b_{i}+W_{hi}h_{t-1}+b_{hi}\right) \end{array}$$

(26) $$\begin{array}{c} y_{t}^{f}=\sigma \left(W_{f}v_{t}+b_{f}+W_{hf}h_{t-1}+b_{hf}\right) \end{array}$$

(27) $$\begin{array}{c} y_{t}^{o}=\sigma \left(W_{o}v_{t}+b_{o}+W_{ho}h_{t-1}+b_{ho}\right) \end{array}$$

(28) $$\begin{array}{c} y_{t}^{c}=\tanh\left(W_{c}v_{t}+b_{c}+W_{hc}h_{t-1}+b_{hc}\right) \end{array}$$

(29) $$\begin{array}{c} c_{t}=y_{t}^{f}⊙h_{t-1}+y_{t}^{i}⊙y_{t}^{c} \end{array}$$

(30) $$\begin{array}{c} h_{t}=y_{t}^{o}⊙\tanh\left(c_{t}\right) \end{array}$$

where $c_{t}$ and $h_{t}$ are the outputs of the LSTM unit and can be passed to the next time step to iterate the aforementioned process. Operator ⊙ stands for element-wise multiplication. W is a weight matrix, with subscripts representing the from–to relationship. For instance, $W_{i}$ is the input gate matrix used for input $v_{t}$. Similarly, $W_{f}$, $W_{o}$, and $W_{c}$ are also matrices for forget, output, and cell to operate on inputs, whereas $W_{hi}$, $W_{hf}$, $W_{ho}$ and $W_{hc}$ are hidden-input, hidden-forget, hidden-output, and hidden-cell matrices used to operate on hidden states. Variables $b_{i},b_{f},b_{o}$, $b_{c}$, etc., are bias vectors.

We use two LSTM layers with 128 units. The output of our first LSTM unit is input to the next unit. In this way, the temporal context of activity data can be learned. The output of LSTM layers is passed to a fully connected layer, as shown in Figure 5. Finally, the output of the fully connected layer is sent to the softmax layer, which employs a softmax function, as described in Equation (17) and produces the vector $q_{C}$ that contains probabilities for each composite activity label. Similar to the atomic activity recognition, the predicted composite activity label is assigned to the one with the highest probability, i.e., $y_{C}\leftarrow \arg\max_{y_{C}=1}^{N}q_{C}$, where $y_{C}$ is the composite activity label, and *N* represents the number of composite activity labels.

### 4.4. Multitask Learning for Atomic and Composite Activities

In this model, all of the three types of activities are trained together. The learning process is described below.

Let $f_{S}\left(S^{\left(k\right)};\theta_{S}\right)$, $f_{B}\left(B^{\left(k\right)};\theta_{B}\right)$, and $f_{C}\left(C^{\left(k\right)};\theta_{C}\right)$ be three classifiers on state activity instance $S^{\left(k\right)}$, behavioral activity instance $B^{\left(k\right)}$, and composite activity instance $C^{\left(k\right)}$, respectively. The terms $\theta_{S}$, $\theta_{B}$, and $\theta_{C}$ represent the parameters of the models of state, behavioral, and composite activities, respectively. All of the classifiers use the softmax function described in Equations (17) and (18) for classification. The cross-entropy loss function is used by these classifiers. The three loss functions $L(f_{S})$, $L(f_{B})$, and $L(f_{C})$ are defined for state, behavioral, and composite activities in Equations (20), (21) and (31), respectively.

(31) $$L\left(f_{C}\right)=-\frac{1}{U}\sum_{u=1}^{U}{y_{C}^{\left(u\right)}}^{T}\log\left(q_{C}^{\left(u\right)}\right)$$

In this equation, the log function is applied to vector $q_{C}^{\left(u\right)}$ and produces the output also in the form of a vector.

Two joint loss functions, $L(f_{SC})$ and $L(f_{BC})$, are computed as follows,

(32) $$L\left(f_{SC}\right)=L\left(f_{S}\right)+L\left(f_{C}\right)$$

(33) $$L\left(f_{BC}\right)=L\left(f_{B}\right)+L\left(f_{C}\right)$$

The goal of the proposed multitask approach is to train the model by minimizing each of the joint loss functions as follows:

(34) $$f_{S},f_{C}\leftarrow \arg\min\;L\left(f_{SC}\right)$$

(35) $$f_{B},f_{C}\leftarrow \arg\min\;L\left(f_{BC}\right)$$

(36) $$f_{C}\left(C;\theta_{C}\right)$$

$\theta_{S}$ and $\theta_{C}$ should be minimized according to the loss function $L(f_{SC})$, and $\theta_{B}$ and $\theta_{C}$ should be minimized according to the loss function $L(f_{BC})$.

(37) $${\theta_{S}}^{*},{\theta_{C}}^{*}\leftarrow \underset{\theta_{S}\theta_{C}}{\arg\min}\;L\left(f_{SC}\right)$$

(38) $${\theta_{B}}^{*},{\theta_{C}}^{*}\leftarrow \underset{\theta_{B}\theta_{C}}{\arg\min}\;L\left(f_{BC}\right)$$

After training, we obtain an optimized set of parameters for functions $f_{S}\left({S;\theta_{S}}^{*}\right)$, $f_{B}\left({B;\theta_{B}}^{*}\right)$, and $f_{C}\left({C;\theta_{C}}^{*}\right)$. When training a multitask model of atomic and composite activities, the backpropagation process in which the loss generated by the classification task of composite activities also affects the parameters of the shared layers of both branches of the model.

## 5. Experimental Setup and Evaluation of Proposed Approaches

In this section, we first present the evaluation metrics that we use to compare different methods. Then, we present the datasets we used in our experiments. Later, we discuss the experimental settings and results for these datasets. We also show a comparative analysis of our approach with other known and state-of-the-art methods.

### 5.1. Evaluation Metrics

The performance of an experiment is estimated from the number of correctly predicted positive labels (TP), the number of incorrectly predicted positive labels (FP), the number of correctly predicted negative labels (TN), and the number of incorrectly predicted negative labels (FN). To indicate the classification performance of the different methods, we use accuracy and F1-score, as mentioned in Equation (39) and Equation (42), respectively. The F1-score is produced for each class; therefore, we compute the mean of F1-scores of all classes and show the results in the form of average F1 (AF1) scores, as described in Equation (43).

(39) $$\mathrm{Accuracy}=\frac{TP+TN}{TP+TN+FP+FN}$$

(40) $$\mathrm{Precision}=\frac{TP}{TP+FP}$$

(41) $$\mathrm{Recall}=\frac{TP}{TP+FN}$$

(42) $$F1\;\mathrm{score}=2*\frac{\mathrm{Precision}*\mathrm{Recall}}{\mathrm{Precision}+\mathrm{Recall}}$$

(43) $$AF1\;\mathrm{score}=\frac{1}{C}\sum_{j=1}^{C}{F1\;\mathrm{score}}_{j}$$

### 5.2. Dataset Description

In our experiments, we used two human activity datasets, namely *CogAge-atomic* and *CogAge-composite*. Both datasets contain time-series data from wearable devices, as shown in Figure 1. Each data instance consists of nine sensor modalities, and each modality contains three sensor channels (x, y, and z). These sensor modalities are mentioned as follows:

1. Smartphone Accelerometer (sp-acc)
2. Smartphone Gyroscope (sp-gyro)
3. Smartphone Gravity (sp-grav)
4. Smartphone Linear Accelerometer (sp-linAcc)
5. Smartphone Magnetometer (sp-magn)
6. Smartwatch Accelerometer (sw-acc)
7. Smartwatch Gyroscope (sw-gyro)
8. Smartglasses Accelerometer (sg-acc)
9. Smartglasses Gyroscope (sg-gyro)

The CogAge-atomic dataset consists of two types of short-term activities, namely state activities and behavioral activities. State activities indicate the state of a subject, e.g., standing, sitting, and walking. Behavioral activities indicate the task a subject is performing, e.g., drinking, cleaning the floor, and opening the door. Data for each atomic activity instance were collected for 5 s. However, due to data-transmission issues, not all channels necessarily have a length of exactly 5 s. Therefore, we decided to use the first 4.5 s of each data instance. The dataset was collected by eight subjects and contains 9029 instances of 61 atomic activities, out of which 886 instances belong to 6 state activities and the remaining 8143 instances belong to 55 behavioral activities.

On the other hand, the CogAge-composite dataset contains the data for composite activities in which a subject performs activities of daily living, e.g., brushing teeth, cleaning room, and preparing food. The length of each composite activity varies according to natural conditions. In our experiments, we used the actual length of a composite activity. The dataset is collected by six subjects, and it contains around 900 instances of seven composite activities.

### 5.3. Recognition of Atomic Activities

We used two approaches to recognize atomic activities, namely the STL and MTL approaches. The data distribution for the experiments of the two approaches is described in the following section.

#### 5.3.1. Data Distribution of Atomic Activities

The data collection for CogAge-atomic was conducted separately for training and testing phases on different days because we wanted to include variations while performing these activities. We name such data settings as train–test settings, in which data of all subjects are included in training and testing datasets. However, these datasets are completely non-overlapping. We used 474 instances for training state activity models and tested these models with 412 instances. To train behavioral activity models, we used 4407 instances and tested these models with 3736 instances.

#### 5.3.2. Description of Existing Approaches on CogAge-Atomic

The following approaches have been implemented on CogAge-atomic by Shirahama [20] and Nisar [19].

##### Codebook Approach for Atomic Activities

This is a kind of bag-of-words approach that creates a feature representation of the data in an unsupervised manner by identifying the characteristic data segments, called codewords, after applying a clustering algorithm. The frequencies of the learned codewords are then used as features to represent the original data sequences. We have applied this approach to recognize atomic activities.

##### CNN Approach for Atomic Activities

A multichannel CNN approach was tested to generate atomic scores for state and behavioral activities.

#### 5.3.3. Single-Task Learning for Atomic Activities

We begin our experiments using the STL method for state and behavioral activities, as described in Section 4.1. Following the method, the multibranch CNN models were trained for state and behavioral activities separately. These models consist of two modules. The first module is called the multibranch-CNN module, as described in Figure 5a, and is identical for both state and behavioral models. In this module, each branch receives the raw data from one of the nine sensor modalities. Each sensor modality has different data dimensions depending upon the sampling rate of the sensors. The raw data of a sensor are inputted to a sensor module, as shown in Figure 4, which involves two consecutive blocks of layers, each of which includes convolutional, RELU activation, and max-pooling layers. The batch normalization is performed on the data prior to sending them to the first block of layers. The outputs produced using the second blocks of all sensor modules are first flattened and then concatenated to construct a big joint vector Φ for all sensor modalities. Table 3 shows the information about the layers and the hyperparameters used in the multibranch–CNN module.

The second module is named either the state-layer or behavioral-layer module and consists of three fully connected layers, including the softmax or output layer. The state STL model contains a multibranch-CNN module and state layers, whereas the behavioral STL model consists of a multibranch-CNN module and behavioral layers. In both models, the respective state or behavioral layers receive the joint vector Φ. The constituent fully connected layers in state or behavioral-layer modules are then used to perform a fusion of the information extracted from all sensor channels. The probabilistic outputs (i.e., atomic scores) for each of the atomic activities are obtained by their respective softmax layers. Table 4 and Table 5 show the information about layers and hyperparameters used in state layers and behavioral layers, respectively. Both STL models used categorical cross-entropy as the loss function.

The models are trained for 1000 epochs using the ADADELTA optimizer [44] with categorical cross-entropy loss functions. The training dataset is used to train the model, and the models are evaluated on the test dataset. The accuracy and average F1-scores are used as evaluation metrics. The models were coded using Python 3.7 and the Pytorch library and trained using a 32 GB RAM machine with an Intel(R) Core(TM) i7-8700 CPU and an Nvidia GTX 1080Ti GPU.

#### 5.3.4. Multitask Learning for Atomic Activities

The multitask models of atomic activities learn state and behavioral activities simultaneously. The architecture of the MTL model consists of a common multibranch-CNN module that is connected to state and behavioral layers, as described in Figure 5b. The raw sensor data of both state and behavioral activities are inputted to the multibranch-CNN module that generates a vector Φ, which is forwarded to both the state and behavioral layers. The softmax functions employed on the last fully connected layers in state- and behavioral-layer modules produce state and behavioral scores, respectively. Categorical cross-entropy functions are used to compute loss for state and behavioral activities. The joint loss $L(f_{A})$ is computed as described in Equation (22). As the number of examples of state and behavioral activities differs in the dataset, we introduced a weighting strategy to adjust the loss accordingly. We used five different options to adjust the weights $w_{B}$ for the behavioral activity loss $L(f_{B})$. Table 6 shows the results of the experiments in which we fixed $w_{S}$ to 1.0 and computed results by using one of the five values for $w_{B}$. The best results were produced using $w_{S}=1.0$ and $w_{B}=0.10$. Therefore, we used this setting in further experiments of MTL atomic models.

The models are trained for 1000 epochs using the ADADELTA optimizer [44]. The training dataset is used to train the model, and the models are evaluated on the test dataset. Accuracy and average F1-scores are used as evaluation metrics. The models were coded using Python 3.7 and the Pytorch library and trained using a 32 GB RAM machine with an Intel(R) Core(TM) i7-8700 CPU and an Nvidia GTX 1080Ti GPU.

### 5.4. Recognition of Composite Activities

Composite activities are also learned using two different approaches, namely the STL and MTL approaches. The training and testing data distribution for the experiments is described in the following section.

#### 5.4.1. Data Distribution of Composite Activities

Similar to the CogAge-atomic dataset, data collection for the CogAge-composite dataset was performed separately for the training and testing phases on different days to capture activity data with variations. Since the number of instances in the composite dataset is comparatively smaller, we trained the models with the following three data settings to show the generalization of the trained models:

- Train–Test: in this setting, the models were trained and evaluated on the data collected separately for the training and testing phases on different days. All subjects were involved in both sessions of data collection. The training dataset consists of 453 instances of composite activities, whereas the testing dataset consists of 449 activity instances. There was no overlapping of instances between the training and testing datasets.
- Leave-one-subject-out cross-validation (LOSO-CV): in this setting, the models were iteratively trained with data from five subjects and then evaluated with data from another single subject. In this configuration, each activity was tested once and included five times in the training set. This approach mitigates potential bias resulting from splitting the data while reducing the variance of the results.
- Holdout: in this setting, the CogAge composite dataset was split into a training and a testing part using a holdout method. The training part included the data from three subjects (specifically, S1, S3, and S4), comprising a total of 481 composite activity instances. The testing part, on the other hand, used the data from the remaining three subjects (S2, S5, and S6), which comprised a total of 421 activity instances.

#### 5.4.2. Description of Existing Approaches on CogAge-Composite

The following approaches were implemented on CogAge-composite by Nisar [19] and Amjad [45].

#####  Max Pooling (MP) and Average Pooling (AP) Approaches for Composite Activities

Max pooling and average pooling are widely recognized techniques for dimensionality reduction in input data while preserving the essential information in the output. In the context of composite activity recognition, these pooling techniques are used to convert a matrix of atomic scores into a feature vector. Both pooling methods produce feature vectors with a dimensionality of 61 for each composite activity instance.

##### Rank Pooling (RP) Approach for Composite Activities

Rank pooling represents a temporal pooling method that gathers relevant information during the execution of a composite activity. This is achieved by training a learning-to-rank model and then using the parameters obtained from this learned model as new features for the composite activity.

#### 5.4.3. Finding Optimal Length of Composite Activities

The CogAge-composite dataset contains composite activity instances with varying lengths. Our initial thought was to fix the instances to a certain length. To find an optimal length, we performed the segmentation of the composite activity instances in three different lengths, i.e., 18, 45, and 90 s. We began our experiments for the recognition of composite activities by training the models with fixed-length instances and produced results for all three segment sizes. Table 7 shows the results on different lengths. We found out that as we increase the lengths, we achieve better results in both single-tasking and multitasking models. Therefore, we decided to use the actual length of each activity instance to obtain the most optimal results in our final experiments. The models trained with actual lengths produced the best results.

#### 5.4.4. Single-Task Learning for Composite Activities

Composite activity-recognition models are trained in a hierarchical approach. The raw sensor data of composite activities are first sent to atomic activity-recognition models to obtain 61 atomic scores, which are then used to recognize composite activity using the LSTM module. However, atomic activity-recognition models require the sensor data to have a certain length. Therefore, composite activity instances are divided into fixed-length segments in the time dimension. As each activity instance has a different length, the number of segments of each instance can be different depending on its length. A time-distributed layer is implemented to generate the sequences of atomic scores for each temporal segment of a composite activity instance. These sequences of atomic scores are forwarded to the LSTM module. The LSTM module contains two LSTM and fully connected layers. The probabilistic scores for each of the seven composite activities are produced by the softmax function.

The generation of atomic scores for the temporal segments of composite activities was achieved by using either of the two atomic recognition approaches, i.e., the STL or the MTL atomic model. In the case of the STL atomic models, the raw sensory data of temporal segments are sent to both state STL and behavioral STL models. The former produces 6 state scores and the latter produces 55 atomic scores. The scores are concatenated and then provided to LSTM modules. In the case of the MTL model, the raw sensory data are sent to the combined MTL atomic model that generates sequences of 61 atomic scores, which are forwarded to the LSTM module.

Table 8 describes the hyperparameters of the LSTM module. The composite models are trained for 1000 epochs using the ADADELTA optimizer [44] with categorical cross-entropy loss functions. The training dataset is used to train the model, and the models are evaluated on the test dataset. Experiments have been performed in holdout and LOSO-CV data settings. Table 9, Table 10 and Table 11 show the results in these three settings, respectively. The accuracy and average F1-scores are used as evaluation metrics. The models were coded using Python 3.9 and the Pytorch library and trained by using a 256 GB RAM machine with an Intel(R) Core(TM) i7-8700 CPU and an Nvidia GeForce RTX 2080 GPU.

#### 5.4.5. Multitask Learning for Composite Activities

**Figure 6:**
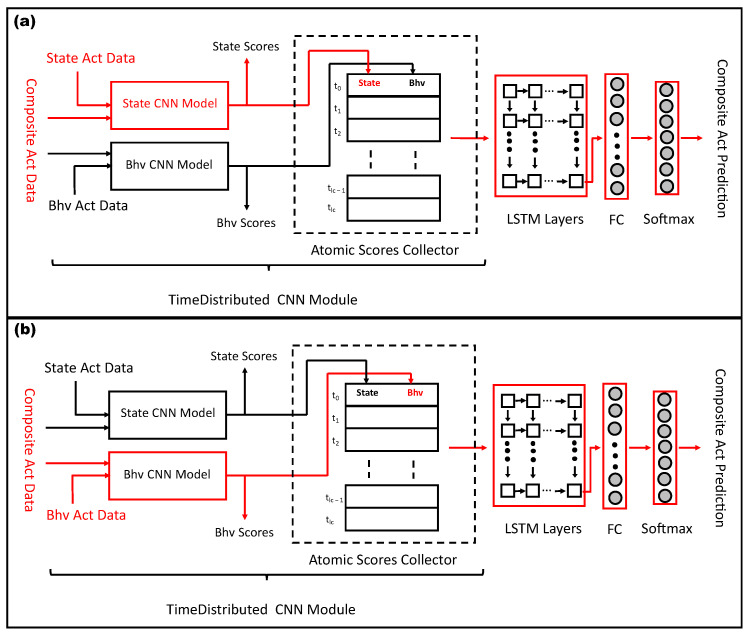
The architecture of the multitask learning model for atomic and composite activities. The first figure (**a**) shows the multitask learning of state and composite activities. The second figure (**b**) shows the multitask learning of behavioral and composite activities.

In the MTL approach, composite activities are trained together with state and behavioral activities. Figure 6 describes the architecture of MTL models of atomic and composite activities. Two MTL approaches were tested in our research study. In the first approach, all three activities were trained together. In each epoch, state, behavioral, and composites were trained. The joint loss was accumulated, and backpropagation was performed to update the weights of all trainable parameters of the model.

In the second approach, two parallel MTL branches were used in the model. State and behavioral activities were trained only with composite activities but not with each other. Both atomic activities have their own exclusive architecture, and they do not share the layers. The training process begins by inputting the raw sensor data of state and behavioral activities to their respective layers. The loss is computed after each batch of instances. Once all the atomic activity instances have been passed through their respective state and behavioral modules, the losses are accumulated by the loss functions $L(f_{S})$ and $L(f_{B})$. Then, the instances of composite activities are segmented along their time dimension. A time-distributed layer receives these temporal segments and passes them to the recently updated state and behavioral modules to generate the sequences of 61 atomic scores. These sequences of atomic scores are forwarded to the LSTM module, which produces the probabilistic scores for each of the seven composite activities. The loss is computed by the function $L(f_{C})$. The accumulated loss of composite activities is used to update the parameters of the LSTM module, as well as the parameters of state and behavioral modules. Thus, in each iteration, not only are the state and behavioral modules updated by their own backpropagation process but their parameters are also influenced by composite activities. Such an MTL architecture produces the best recognition scores for the three types of activities.

The composite models are trained for 1000 epochs using three ADADELTA optimizers [44] with categorical cross-entropy loss functions. The ${\mathrm{optimizer}}_{st}$ and the ${\mathrm{optimizer}}_{bh}$ are used to update the trainable parameters of state and behavioral modules. The ${\mathrm{optimizer}}_{comp}$ is used to update the parameters of the whole model. The training datasets of the three types of activities are used to train the model, and they are evaluated on their test datasets. For composite activities, the experiments have been performed in both holdout and LOSO-CV data settings. Table 10 shows the results obtained from holdout settings. Table 12 shows the result after each pass of the MTL method obtained from LOSO-CV data settings. It can be seen that for some subjects, the obtained results are comparatively better than the others. Table 11 also shows the results in LOSO-CV settings, along with other methods. The accuracy and average F1-scores are used as evaluation metrics. The models were coded using Python 3.9 and the Pytorch library and trained using a 256 GB RAM machine with an Intel(R) Core(TM) i7-8700 CPU and an Nvidia GeForce RTX 2080 GPU.

### 5.5. Results and Discussion

We have summarized all the results in three tables, Table 9, Table 10 and Table 11. The tables show the methods used for atomic and composite activities and the results obtained with these methods for each type of activity. Table 9 shows the results obtained in train–test data settings for the composite activities. The first two rows list the results of state and behavioral atomic activities obtained using the codebook approach. The same rows list the results of the composite activities obtained using the rank pooling approach and also by using a combination of the max, average, and rank pooling approaches. The third row shows the results of atomic activities obtained with the CNN approach and the results of composite activities obtained using the rank pooling approach. The fourth row of the table shows the results of atomic and composite activities obtained with STL approaches. The fifth row shows the results of atomic activities obtained with the MTL approach, while the results of composite activities are presented with the STL time-distributed LSTM model. The sixth row shows the results obtained with the MTL model for all three activities. The seventh and final row shows the results of the MTL models where the composite activities were trained with state and behavioral activities separately.

The proposed MTL methods yielded the results of all activities with an improvement of almost 3%, 2%, and 6% for the recognition tasks of state, behavioral, and composite activities, respectively, as compared to the previous methods applied to these datasets. It can also be observed that the MTL method applied to cooperative tasks like state and composite, and behavioral and composite, yields the best performance overall.

Table 10 shows the results obtained in the holdout data settings of the composite activities. It is a subject-independent setting to show the generalizability of our proposed methods. The format of the table is similar to Table 9. Likewise, the proposed methods also produce the best results in this data setting. An improvement of almost 2.5%, 2%, and 13% can be seen as compared to the previous methods in the recognition tasks of state, behavior, and composite activities respectively.

Table 11 shows the results obtained in another subject-independent, i.e., LOSOCV, data settings of the composite activities. The format of the table is similar to the two previous tables, and the proposed methods also yield the best results as compared to the previous methods. An improvement of almost 1.5%, 2%, and 14% can be observed for the recognition tasks of state, behavior, and composite activities respectively.

Table 12 shows the results obtained after each step in the LOSOCV data settings using the MTL method. It can be observed that the composite activity data of each subject have significant variations for two main reasons: firstly, the individual’s own way of performing the same activity, and secondly, the settings in which the data collected were different according to the subject’s household arrangement. Such variations influenced the results of composite activities within the range of almost 20% accuracy. However, due to the generalizability of our proposed method, the average accuracy is still significantly higher than the previous methods.

To determine which tasks are better learned with the single-task learning paradigm and which tasks should be better learned with the multitask learning paradigm, we conducted extensive experiments in which one category of activities was learned with the single-task learning approach and two other categories of activities were learned with the multitask learning approach. These experiments were conducted in three sub-settings. In the first sub-setting, we trained the models for the atomic activities independently. Then, using the pre-trained models, we performed the training for behavioral and composite activities together. In the second setting, the behavioral activities were learned individually, but the state and composite activities used the pre-trained model of behavioral activities during their training phase. In the third sub-setting, the state and behavioral activities were learned together, while the composite activities were trained using the pre-trained models of the two atomic activities. The results are shown in Table 13.

#### Highlights of the Results

- We have presented and tested with STL and MTL approaches to recognize activities of daily living. Table 9, Table 10 and Table 11 show the results obtained from different methods.
- All of our proposed approaches outperform the previous methods implemented on the CogAge-atomic and Cogage-composite datasets.
- MTL methods improve the results when they are implemented on cooperating tasks. The performance of the MTL methods deteriorates when they are implemented for competing or conflicting tasks.
- State activities are learned better in the STL method than in the MTL method with behavioral activities. It is observed that the performance of state activities deteriorates by 17% to 21% when they are learned together with behavioral activities. The main reason for this high ratio of misclassification is that behavioral activities are acquired when a person is in one of the state postures; therefore, each behavioral activity instance contains not only the data of the behavioral task but also the data of any of the state postures. This phenomenon causes conflict in classifying state activity, especially when it comes to classifying sitting and standing state activities (see Table 14) because the data for most of the behavioral activities were acquired in these two state postures.
- Behavioral activities also do not improve when learned along with state activities. However, they do not show a striking deterioration in performance when learned together with state activities. This is because their dataset is very large compared to the state activities.
- Composite activities are better learned when they are learned together with either state or behavioral activities or both.
- The best results for all three activities are obtained when state and behavioral activities are learned with composite activities but not with each other.
- As composite activities also contain data from different behavioral and state activities, then why are the state activities not confused or misclassified when trained with composite activities? The possible reason could be that each segment of a composite activity contains different behavioral and state tasks and is not fixed to only one state or behavioral task for the entire duration of a segment of the composite activity. This phenomenon is different from what we observed when training the state and behavior together, because in this situation, the same pose is maintained for the entire length of the segment. Therefore, composite activity data not only help in obtaining good accuracy for the state but also in the joint training of state and behavior, in which the accuracy of composite activities is improved by using LSTM models.

## 6. Conclusions and Future Work

In this paper, we proposed our multitask learning framework to jointly solve atomic and composite activity-recognition tasks. For atomic activity recognition, our proposed method used a multibranch CNN to find deep features automatically; these characteristics are discriminating and task-specific. For the composite activity recognition, the features after the use of the CNN are gathered using a TimeDistributed layer before inputting them into an LSTM network. In this way, we can completely use the temporal context of activity data. We presented several architectures for the three tasks in which they have a shared structure (i.e., the CNN, TimeDisributed, and LSTM layers), which can benefit all tasks and improve the generalization ability of our multitask learning methods. We evaluated our approach on two datasets, i.e., CogAage-atomic and CogAge-composite, and the experimental results showed that the proposed model was able to exhibit a competitive performance in both atomic and composite activity recognitions as compared to the single-task learning methods.

In addition to proposing the multitask methods, we described the problem of task compatibility, as it pertains to multitask learning. We performed experiments to determine which tasks should be trained jointly and which tasks should be trained separately in a given setting.

We have observed that the state activities are learned better when they are learned along with composite activities, while the models of behavioral activities produce the best results when they are learned alone. However, when behavioral activities are learned along with composite activities, they still produce results close to their best results. Our third observation is that the models of composite activities produce the best results when they are learned together with state and behavioral activities. So we experimented with a framework in which two parallel multitask learning sequences are performed. In the first sequence, we trained state and composite activities together, and in the second sequence, we trained behavioral and composite activities together. This proposed framework produced the best results for all activities.

The proposed approach yielded improved results as compared to the other state-of-the-art methods on the CogAge-atomic and CogAge-composite datasets. However, it has one limitation: the accuracy of behavioral activities is still under 75%, which seems to be unsatisfactory. The first reason could be the diversity of the behavioral activities, as they were performed in a variety of ways. The second possible reason could be their conflict with the other activities, which might lead to comparatively low results, as the instances of behavioral activity contain information not only about the behavior but also about the state of the subject. Therefore, as a future work, a disentanglement procedure can be used to separate the behavior from the state. Generative adversarial networks can be useful for such disentanglement [46]. The behavior-specific data should then be used for improved classification. Another approach that can help to improve the recognition mechanism is the transformer-based approach that relies on self-attention-based mechanisms [47]. Such models have been useful in learning long sequences in natural language processing, so they should yield good results for long sequences of time-series data acquired from wearable motion sensors as well [48,49]. Therefore, these approaches should be used to recognize behavioral and composite activities.

## Figures and Tables

**Table 3:** Architecture and hyperparameters of the multibranch convolutional module, containing the sequence of layers, the number of convolutional filters and kernel sizes, pooling, and stride sizes used in the model.

Multibranch CNN Module: Architecture and Hyper Parameters				
		Sensors		
Layer	Information	sp-acc, sp-gyro	sp-magn	sg-acc
		sp-grav, sp-linAcc	(sw-acc, sw-gyro)	sg-gyro
Input	Size	X, 3, 900	X, 3, 450	X, 3, 80
Batch normalization		Yes	Yes	Yes
Conv 1	Filters	16	16, (32)	16
	Kern size	64	32	8
	Activation	reLU	reLU	reLU
Max Pool 1	Pool, Stride	16, 4	16, 2	8, 2
Conv 2	Filters	32	32, (64)	16
	Kern size	128	64	8
	Activation	reLU	reLU	reLU
Max Pool 2	Pool, Stride	16, 4	16,2	8,2
Flattening and Concatenation of All Branches				

**Table 4:** Architecture and hyperparameters of state layers, containing the sequence of layers, number of units, activation functions, optimizer, and the loss function used in the model.

State Layers: Architecture and Hyper Parameters				
		Sensors		
Layer	Information	sp-acc, sp-gyro	sp-magn	sg-acc
		sp-grav, sp-linAcc	(sw-acc, sw-gyro)	sg-gyro
FC 1	Units State	In: 10,752, Out: 256		
	Activation		reLU	
Dropout	Rate		0.3	
FC 2	Units State		In: 256, Out: 64	
	Activation		reLU	
Dropout	Rate		0.3	
Output	Units State		In: 64, Out: 6	
	Activation		softmax	
Optimizer: Adadelta, Learning rate: 0.0001				
Loss function: Categorical Cross Entropy				

**Table 5:** Architecture and hyperparameters of the behavioral layers, containing the sequence of layers, number of units, activation functions, optimizer, and the loss function used in the model.

Behavioral Layers: Architecture and Hyper Parameters				
		Sensors		
Layer	Information	sp-acc, sp-gyro	sp-magn	sg-acc
		sp-grav, sp-linAcc	(sw-acc, sw-gyro)	sg-gyro
FC 1	Units Bhv		In: 10,752, Out: 512	
	Activation		reLU	
Dropout	Rate		0.3	
FC 2	Units Bhv		In: 512, Out: 256	
	Activation		reLU	
Dropout	Rate		0.3	
Output	Units Bhv		In: 256, Out: 55	
	Activation		softmax	
Optimizer: Adadelta, Learning rate: 0.0001				
Loss function: Categorical Cross Entropy				

**Table 6:** Results (accuracy) obtained from multitask learning models of state and behavioral activities using different weighting strategies. The results of the experiments are obtained by fixing the weight for state activities, $w_{S}$ to 1.0, and computed results using one of the five values for the weight of behavioral activities $w_{B}$. The best results were produced using $w_{S}=1.0$ and $w_{B}=0.10$. Therefore, we used this setting in further experiments of MTL atomic models.

Comparison of Results with Different Values of Bhv Weights ($w_{B}$)					
Activity	Bhv Weights				
	1.0	0.10	0.01	0.001	0.0001
State	73.54	75.00	72.82	74.76	72.57
Behavioral	73.55	73.34	71.63	71.09	69.14

**Table 7:** Comparative results (accuracy) obtained from different methods, including multitask learning for atomic and composite activities and the single-task learning of composite activities using different lengths.

Comparison of Results Using Different Lengths of Composite Activity Instances					
Length	MTL			STL	
	State	Bhv	Composite	Holdout	Train-Test
18 s	64.56	30.61	80.63	39.90	77.92
45 s	44.66	32.35	81.85	42.38	81.47
90 s	69.18	67.01	82.83	46.31	83.21
Actual	**72.82**	**71.63**	**92.87**	**54.87**	**90.89**

**Table 8:** Architecture and hyperparameters of the LSTM module, containing the sequence of layers, number of units, activation functions, optimizer, and the loss function used in the model.

LSTM Module: Architecture and Hyper Parameters	
Layer	Information
Timedistributed-	Input: Sensors’ data of all ($lc^{k}$) segments of an instance $C^{\left(k\right)}$
State model	Output: An array of $lc^{k}$ score-vectors; each vector contains 6 state scores
Timedistributed-	Input: Sensors’ data of all ($lc^{k}$) segments of an instance $C^{\left(k\right)}$
Behavioral model	Output: An array of $lc^{k}$ score-vectors; each vector contains 55 behavioral scores
Concatenation of state and behavioral score-vectors	
LSTM layers	Input size: 61
	Hidden units: 128
	Num of layers: 2
Output	Input size: 128, Output size: 7
Optimizer: Adadelta, Learning rate: 0.00001	
Loss function: Categorical Cross-Entropy	

**Table 9:** Comparison of results of state and behavioral activities obtained from different methods. The results have been compared using three methods, “codebook and SVM approach”, “multibranch CNN using single-task learning(STL)”, and “multibranch CNN architecture using multitask learning(MTL)”, as explained in the methodology section. The atomic and composite models were trained and evaluated using train–test data settings.

Recognition of State, Behavioral & Composite Activities (Train-Test)							
Method		State		Behavioral		Composite	
Atomic	Composite	Acc	AF1	Acc	AF1	Acc	AF1
Codebook	RP	88.58	88.22	68.23	67.92	81.49	80.92
Codebook	RP + MP + AP					88.49	87.98
CNN	RP	92.41	92.32	71.83	71.68	82.89	82.57
STL Multibranch-CNN	STL Td LSTM	93.69	92.71	73.66	73.22	91.31	90.89
MTL Multibranch-CNN	STL Td LSTM	77.43	77.48	72.03	71.30	91.98	91.79
MTL_Atomic_Composite		74.27	73.25	72.19	71.66	92.87	92.32
MTL_(State-Comp, Bhv-Comp)		**95.15**	**95.07**	**73.93**	**73.38**	**93.99**	**93.76**

**Table 10:** Comparison of results of state and behavioral activities obtained from different methods. The results have been compared using three methods: codebook and SVM approach, multibranch CNN using single-task learning (STL), and multibranch CNN architecture using multitask learning (MTL), as explained in the methodology section. The atomic models were trained and evaluated using train–test settings, whereas composite activity models were trained and evaluated using train–test data settings.

Recognition of State, Behavioral and Composite Activities (Holdout)							
Method		State		Behavioral		Composite	
Atomic	Composite	Acc	AF1	Acc	AF1	Acc	AF1
Codebook	RP	88.58	88.22	68.23	67.92	61.48	60.91
Codebook	RP + MP + AP					63.64	63.65
CNN	RP	92.41	92.32	71.83	71.68	54.98	54.31
STL Multibranch-CNN	STL Td LSTM	93.69	92.71	**73.66**	**73.22**	59.62	53.61
MTL Multibranch-CNN	STL Td LSTM	77.43	77.48	72.03	71.30	55.11	51.76
MTL_Atomic_Composite		76.94	76.33	71.63	71.42	**76.01**	**75.80**
MTL_(State-Comp, Bhv-Comp)		**94.90**	**94.85**	**73.48**	**73.10**	70.55	69.01

**Table 11:** Comparison of results of state and behavioral activities obtained from different methods. The results have been compared using three methods: codebook and SVM approach, multibranch CNN using single-task learning(STL), and multibranch CNN architecture using multitask learning(MTL) as explained in the methodology section. The atomic models were trained and evaluated using train-test settings whereas composite activity models were trained and evaluated using LOSO-CV data settings.

Recognition of State, Behavioral & Composite Activities (LOSO-CV)							
Method		State		Behavioral		Composite	
Atomic	Composite	Acc	AF1	Acc	AF1	Acc	AF1
Codebook	RP	88.58	88.22	68.23	67.92	64.58	62.23
Codebook	(RP + MP + AP)	88.58	88.22	68.23	67.92	68.65	64.39
CNN	RP	92.41	92.32	71.83	71.68	56.80	52.85
STL Multibranch-CNN	STL Td LSTM	93.69	92.71	73.66	73.22	77.37	76.60
MTL Multibranch-CNN	STL Td LSTM	77.43	77.48	72.03	71.30	72.22	70.11
MTL_Atomic_Composite		75.00	74.96	72.18	71.66	**82.31**	80.67
MTL_(State-Comp, Bhv-Comp)		**93.77**	**93.07**	**73.73**	**73.38**	81.28	**80.72**

**Table 12:** Comparison of results obtained from multitask learning method. The two-branch MTL model was jointly trained for state and composite activities in one branch and behavioral and composite activities in the second branch. The results were obtained in the LOSO-CV settings of the composite activity dataset.

LOSO-CV: Multitask Learning for State, Bhv and Composite Activities			
Pass	State	Behavioral	Composite
Pass-1	93.45	73.82	90.00
Pass-2	95.39	73.93	71.93
Pass-3	92.72	73.56	77.63
Pass-4	93.20	74.44	81.51
Pass-5	94.16	72.79	90.71
Pass-6	93.69	73.85	75.91
Average	93.77	73.73	81.28

**Table 13:** Performance comparison of multitask learning for two types of activities.

Activity	State	Behavioral	Composite
State + Behavioral	77.43	72.03	-
State + Composite	95.17	-	92.43
Behavioral + Composite	-	73.92	93.32

**Table 14:** Confusion matrix of state activities obtained from the MTL model of state and behavioral activities. The values in the circles show that most of the misclassification occurred in sitting and standing state activities.

State Activities—Confusion Matrix—MTL State and Behavioral						
Activities	Bending	Lying	Sitting	Squatting	Standing	Walking
Bending	69	0	0	0	0	0
Lying	6	63	0	0	0	0
Sitting	43	2	19	3	0	0
Squatting	17	0	2	48	0	0
Standing	43	0	0	0	27	0
Walking	5	0	0	0	0	65

## Data Availability

The dataset used in this study is available upon approval of a research request to M.A.N.
